# The role of public and private natural space in children's social, emotional and behavioural development in Scotland: A longitudinal study

**DOI:** 10.1016/j.envres.2017.07.038

**Published:** 2017-10

**Authors:** Elizabeth A. Richardson, Jamie Pearce, Niamh K. Shortt, Richard Mitchell

**Affiliations:** a Centre for Research on Environment, Society and Health, Research Institute of Geography and the Lived Environment, University of Edinburgh, Drummond Street, Edinburgh EH8 9XP, United Kingdom; b Centre for Research on Environment, Society and Health, Institute of Health and Wellbeing, University of Glasgow, 1 Lilybank Gardens, Glasgow G12 8RZ, United Kingdom

**Keywords:** CI, Confidence Interval, GUS, Growing Up in Scotland survey, IQR, Interquartile Range, SDQ, Strengths and Difficulties Questionnaire, SF-12, The 12-item Short Form health questionnaire, SGM, Scotland's Greenspace Map, SIMD, Scottish Index of Multiple Deprivation, Nature, Children, Social development, Emotional development, Behavioural development, Strengths and Difficulties Questionnaire

## Abstract

**Introduction:**

Poor mental health in childhood has implications for health and wellbeing in later life. Natural space may benefit children's social, emotional and behavioural development. We investigated whether neighbourhood natural space and private garden access were related to children's developmental change over time. We asked whether relationships differed between boys and girls, or by household educational status.

**Methods:**

We analysed longitudinal data for 2909 urban-dwelling children (aged 4 at 2008/9 baseline) from the Growing Up in Scotland (GUS) survey. The survey provided social, emotional and behavioural difficulty scores (Strengths and Difficulties Questionnaire (SDQ)), and private garden access. Area (%) of total natural space and parks within 500 m of the child's home was quantified using Scotland's Greenspace Map. Interactions for park area, total natural space area, and private garden access with age and age^2^ were modelled to quantify their independent contributions to SDQ score change over time.

**Results:**

Private garden access was strongly related to most SDQ domains, while neighbourhood natural space was related to better social outcomes. We found little evidence that neighbourhood natural space or garden access influenced the trajectory of developmental change between 4 and 6 years, suggesting that any beneficial influences had occurred at younger ages. Stratified models showed the importance of parks for boys, and private gardens for the early development of children from low-education households.

**Conclusion:**

We conclude that neighbourhood natural space may reduce social, emotional and behavioural difficulties for 4–6 year olds, although private garden access may be most beneficial.

## Highlights

• Children with gardens had better social, emotional and behavioural scores.
• Children with more neighbourhood natural space had better social skills.
• Developmental trajectories did not differ with natural space availability.

## 1 Introduction

Poor mental health in childhood has implications for health and wellbeing in later life, and presents a considerable burden for families and wider society. In the short term, for example, school attainment may be impaired (Trout et al., 2003), while in the longer term persistent mental health issues, higher mortality rates and wider inequalities may result (Dube et al., 2003, Jokela et al., 2009). Recent decades have seen substantial increases in the prevalence of childhood social, emotional, and behavioural problems (Layard and Dunn, 2009). To address this upward trend, and the consequent growing societal burden now and in the future, it is imperative to identify the determinants of these childhood problems. Individual, family, and household characteristics contribute, but they do not explain all of the variation in risk (Bradshaw and Tipping, 2010, Wilson et al., 2012). Environmental influences – including noise (Forns et al., 2015), air pollution (Forns et al., 2015), and a lack of contact with natural space (Amoly et al., 2014) – have also been identified as possible risk factors for poor mental health in childhood.

Our study examines the role that natural space might play in children's development. Louv (2005) argued that there are substantial negative effects of ‘alienation’ from nature, and that these may be the root cause of increases in childhood developmental problems. Today's children spend less time outdoors in nature than previous generations (Gaster, 1991), and tend to be less physically active and more obese (Anon, 2013). Urbanisation, increasingly indoor pastimes, and parental concerns about safety may all have contributed to declining childhood nature experiences (Strife and Downey, 2009, Valentine and McKendrick, 1997).

A growing body of research has found that children who live or spend time in more natural surroundings typically have fewer social, emotional and behavioural problems than those in less green settings (Amoly et al., 2014, Faber Taylor and Kuo, 2009). A number of causative mechanisms have been suggested. Firstly, experiences of natural environments may directly restore a child's attention by giving fatigued cognitive processes the opportunity to rest (“Attention Restoration Theory”, Kaplan and Kaplan, 1989). In a US study schoolchildren who moved to more natural settings exhibited greater improvement in their attention levels than others (Wells, 2000), and in Barcelona, children with greener surroundings had better memory and attention levels (Dadv and et al., 2015). Secondly, natural environments may support stress reduction through favourable physiological responses (“Psychoevolutionary Theory”; Ulrich, 1983). Wells and Evans (2003) reported that levels of nearby nature buffered the impact of stressful life events on schoolchildren. Thirdly, natural environments may increase opportunities for play (Almanza et al., 2012), which in natural settings is typically more creative, adventurous, social, and challenging than play elsewhere (Hart, 1979). Indeed, increased usage of green space in urban areas has been linked to improved health and wellbeing in Scottish schoolchildren (McCracken et al., 2016). Fourthly, natural space availability may indirectly affect the child via effects on their carer. Exposure to natural spaces has been linked with better mental health in adulthood (Hartig et al., 2014), and the carer's mental health can influence early childhood development (Marryat and Martin, 2010).

Research into the potential role of nature in childhood development focusses on school-aged children. However, considering younger children is critical because of the important capabilities in exploration, imagination, socialisation, and control that develop through increasingly independent play at younger ages (Bee, 1992, Erikson, 1963). Further, different types of natural space may be more or less beneficial for children's development, but this has been little researched. The developmental benefits of play are optimised when children are able to explore the space and construct things (e.g., shelters) with minimal adult intrusion, and to interact with others (Hart, 1979). Expansive public spaces may therefore be more beneficial (e.g. parks rather than private gardens or overall natural space). Indeed, Lithuanian 4–6 year olds had fewer emotional and behavioural problems if they had better availability of parks nearby, although these problems were not related to overall green space (Balseviciene et al., 2014). Alternatively, play with minimal supervision – particularly for young children – may satisfy parents’ safety concerns more if it takes place in a private garden rather than a public space. In this case having access to a private garden may be more important than natural space in the neighbourhood: 3–7 year old children in England with access to a garden had lower levels of social, emotional and behavioural problems, but neighbourhood green space was unrelated (Flouri et al., 2014).

Evidence for the determinants of early childhood development problems is urgently needed to inform public health interventions. Here we expand the evidence base by investigating whether social, emotional and behavioural development for young children (age 4 at baseline) is better for those with more natural space around their homes, and particularly more public park space, or whether access to a private garden is more important. We explore differences by sex and household socioeconomic status, given known differences in how these groups use and are affected by their local environments (Cleland et al., 2010, de Vries et al., 2003).

## 2 Methods

### 2.1 Study population

We analysed data from the Growing Up in Scotland (GUS) survey (Scottish Centre for Social Research, 2012). GUS's nationally-representative birth cohort sample was selected in 2005/2006 (*n* = 5217 achieved interviews) from families with babies of approximately 12 months in receipt of child benefits (97% of families with children in Scotland) – at that time a non-means-tested benefit paid to carers of children under 16 – and was followed up annually thereafter. Sampling stratification ensured a representative selection of areas of differing socioeconomic status within each local authority (Wilson et al., 2012). We selected respondents from wave five (age 5, 2009/2010; *n* = 3833 achieved interviews) because these children's home postcodes were available through a secure setting. There are over 200,000 postcodes in Scotland, each representing approximately 15 households. We selected the 2909 children (76%) living in areas of Scotland covered by the urban natural space data (see Section 2.3) at wave five. The child's wave four (age 4) and six (age 6) survey data could be included if they had been living at their wave five address then (i.e., non-movers), resulting in an additional 2650 wave four and 2482 wave six observations.

### 2.2 Outcome variables

Social, emotional and behavioural difficulties were assessed using the 25-item Strengths and Difficulties Questionnaire (SDQ; Goodman, 1997) in waves four, five and six. The SDQ – a behavioural screening tool designed for children between 3 and 16 years old – has been widely used internationally, owing to its good psychometric properties and clinical utility (Theunissen et al., 2015). The questionnaire was self-completed by the main carer, usually the mother.

For each SDQ domain - Hyperactivity Problems, Emotional Problems, Peer Problems, Conduct Problems, and Prosocial Behaviour - the respondent was asked whether each of five items (Table 1) was ‘Not true’, ‘Somewhat true’ or ‘Certainly true’ of the child's behaviour over the last six months. Responses were scored 0, 1, or 2, with 2 being the most negative (or most positive, in the case of Prosocial Behaviour). The scores were summed to give a domain score of 0–10, and a Total Difficulties score (ranging 0–40) was calculated by summing all domains except Prosocial Behaviour. Higher scores indicated worse problems (opposite for Prosocial Behaviour).

**Table 1:** Items within the Strengths and Difficulties Questionnaire (SDQ) domains.

SDQ domain	Items
Hyperactivity Problems	Restless, overactive, cannot stay still for long
	Constantly fidgeting or squirming
	Easily distracted, concentration wanders
	Thinks things out before acting
	Sees tasks through to the end, good attention span
Emotional Problems	Often complains of headaches, stomach aches or sickness
	Many worries, often seems worried
	Often unhappy, downhearted or tearful
	Nervous or clingy in new situations, easily loses confidence
	Has many fears, is easily scared
Peer Problems	Rather solitary, tends to play alone
	Has at least one good friend
	Generally liked by other children
	Picked on or bullied by other children
	Gets on better with adults than with other children
Conduct Problems	Often has temper tantrums or hot tempers
	Generally obedient, usually does what adults request
	Often fights with other children or bullies them
	Often lies or cheats
	Steals from home, school or elsewhere
Prosocial Behaviour	Considerate of other people's feelings
	Shares readily with other children
	Helpful if someone is hurt, upset or feeling ill
	Kind to younger children
	Often volunteers to help others (parents, teachers, other children)

### 2.3 Natural space measures

We quantified the area of public parks and total natural space around each child's home, using 2011 data (at around wave six). We obtained ‘Scotland's Greenspace Map’ (SGM; Greenspace Scotland, 2011) in geographical information system shapefile format. The SGM study area covered settlements in Scotland with populations greater than 3000 in 2001, plus a 500 m buffer. Each polygon of a high resolution (centimetre-accuracy) vector map product (Ordnance Survey's MasterMap) had been manually classified into types (e.g., park, playing field, church yard, or school ground) using aerial photography. We used the primary land use class only, unless ‘public park’ had been identified as a secondary land use (all were included as public park).

We found some incomplete mapping and overlapping polygons in the SGM dataset. We identified postcodes within the study area that did not have natural space mapped within 30 m, and used aerial photography (Google Maps) to verify this. We found 740 postcodes (0.5% of a total of 157,282) with incomplete natural space mapping, and excluded these from the analysis. The full list of excluded postcodes is available as Supplemental Material. Overlapping polygons were identified in 1909 locations (mean overlap size 106 m^2^). One overlapping portion in each case was deleted to prevent artificially-inflated area calculations. Portions of parkland were preferentially retained in the dataset (e.g., if woodland overlapped with park the woodland polygon was deleted).

Agricultural land and some open water had not been mapped in the SGM. As both land uses could provide nature experiences we augmented the dataset accordingly. Agricultural areas were extracted from the European Environment Agency's 2006 CORINE dataset (CORINE classes 12–22) and added to SGM if they occurred in unmapped parts of the study area. Open water areas not already mapped in the study area were added from Ordnance Survey's VectorMap product.

We calculated the area of public parks and total natural space within 500 m (Euclidean distance) of each child's postcode, representing a young child's walk of approximately 10 min. Total natural space included all public and private natural surfaces – vegetation, water, sand, mud and rock – and included private gardens. Geoprocessing was conducted using ArcMap 10.1 software (ESRI, Redlands, CA).

Whether the child had access (sole or shared) to a private garden was obtained from the survey data.

### 2.4 Covariates

We adjusted for possible confounders of the relationship between the child's SDQ scores and natural space (Bradshaw and Tipping, 2010, Pachter et al., 2006, Wilson et al., 2012). Child covariates were sex, age (decimal years, centred at the grand mean of 4.85), age^2^ (to capture non-linear temporal trends; mean-centred), and hours of screen time per day (constrained to a maximum of 8 h to address some erroneous values). Household covariates were highest educational attainment (degree or equivalent, vocational qualification below degree, Higher/Standard grades or equivalent, and other or no qualifications), equivalised annual income (continuous), and the carer's mental component summary score on the SF-12 questionnaire (0–100, with higher score indicating better mental health; Ware et al., 1996). Neighbourhood-level disadvantage was measured using national-level quintiles of the 2009 Scottish Index of Multiple Deprivation (SIMD; Scottish Government, 2009), for the child's residential ‘datazone’ (administrative unit containing 500–1000 residents). Missing values for the dependent and independent variables (see Table 2) were imputed (five imputations) using multiple imputation with chained equations in Stata SE/14.0 (StataCorp, College Station, TX).

**Table 2:** Individual, household and neighbourhood characteristics for the 2909 children in the sample, as at wave five.

Level	Characteristic	Mean (95% CI)	Count (%)
Individual	Age (years)	4.85 (4.85, 4.85)	
	Sex		
	Male		1478 (51)
	Female		1431 (49)
	Screen time (hours)	2.38 (2.32, 2.44)	
	Missing		708 (24)
	SDQ score:		
	Hyperactivity Problems	3.69 (3.60, 3.77)	
	Missing		32 (1)
	Emotional Problems	1.25 (1.19, 1.30)	
	Missing		24 (1)
	Peer Problems	1.05 (1.00, 1.10)	
	Missing		23 (1)
	Conduct Problems	1.70 (1.65, 1.76)	
	Missing		23 (1)
	Total Difficulties	7.67 (7.50, 7.84)	
	Missing		37 (1)
	Prosocial Behaviour	8.22 (8.16, 8.28)	
	Missing		24 (1)
	Dental caries		
	Yes		220 (8)
	No		2689 (92)
Household	Highest educational attainment:		
	Degree		1108 (38)
	Vocational qualification		1109 (38)
	Higher/Standard grade		406 (14)
	Other/no qualification		135 (5)
	Missing		151 (5)
	Access to a garden?		
	Yes		2740 (94)
	No		166 (6)
	Missing		3 (0)
	Equivalised household income (£000 s)	24.08 (23.61, 24.55)	
	Missing		157 (5)
	Carer's mental health score (SF12)	50.34 (50.00, 50.69)	
	Missing		20 (1)
Neighbourhood	SIMD quintile:		
	Quintile 1 (least deprived)		741 (25)
	Quintile 2		424 (15)
	Quintile 3		480 (17)
	Quintile 4		608 (21)
	Quintile 5 (most deprived)		656 (23)
	Total natural space (% within 500 m)	63.07 (62.58, 63.55)	
	Park space (% within 500 m)	4.69 (4.44, 4.94)	

### 2.5 Statistical analyses

We ran random-intercept repeated-measures linear models, with waves nested within individuals nested within the survey's Primary Sampling Units. We entered percentage total natural space, percentage park space, and garden access as main effects, to quantify whether they had a consistent association with each SDQ domain across the study period. We also included their interactions with age and age^2^, to quantify their contributions to SDQ score trajectories between ages 4 and 6. To assess their independent contributions to any relationship found, park space was modelled concurrently with total natural space, even though the latter included the former. On average only 3% (median) of total natural space was park, and the areas of both correlated weakly (r = 0.15). To capture the individual children's SDQ score trajectories we tested the inclusion of a random slope for age, but as this did not alter the results these models are not presented.

We ran the models in MLwiN (Rasbash et al., 2009) using Stata's runmlwin routine (Leckie and Charlton, 2013). First-order marginal quasi-likelihood estimates were used as initial values for a Markov Chain Monte Carlo estimation (Browne, 2016). The models used a Markov chain length of 40,000, with orthogonal parameterisation and hierarchical centring at the individual level. We did not weight our urban subsample as we were not seeking to produce representative estimates for the wider population. After running whole sample models we stratified by sex and by household educational attainment (degree/equivalent versus lower).

Our models may have been subject to residual confounding, particularly if we had not fully captured socioeconomic disadvantage. We therefore chose to also model a socially-patterned control outcome without a plausible link to natural space: whether the child had fillings (treatment for dental caries) by wave five. Among our sample, prevalence rates of dental caries were highest for children from households with the lowest educational attainment (10%) and the most deprived areas (10%), and lowest for those from the most educated households (5%) and the least deprived neighbourhoods (7%).

To retain the full amount of information in the SDQ scores we modelled them as continuous variables. To test the sensitivity of the results to this decision we also ran logistic versions of the main models, with the scores dichotomised into normal/borderline and abnormal, as defined by Goodman (1997). Abnormal scores were ≥17 for Total Difficulties, ≥4 for Conduct Problems, and Peer Problems, ≥5 for Emotional Problems, ≥7 for Hyperactivity Problems, and ≤4 for Prosocial Behaviour.

## 3 Results

### 3.1 Descriptive statistics

The children had an average of 63% natural space within 500 m of their home postcode (Table 2). A small proportion of this (7%) was public park. We present model coefficients for an interquartile range (IQR) increase in each type: 16.2% points for total natural space and 6.8 for public parks. Most (94%) of the children had access to a garden. Those without access to a garden had significantly less total natural space around their homes (54.8%, 95% confidence interval 52.3–57.4) than those with garden access (63.6%, 63.1–64.1; t = 8.3, d.f. = 2904, p < 0.001), and significantly more park space (6.5% 5.3–7.7%) c.f. (4.6% 4.3–4.8%; t = −3.4, d.f. = 2904, p < 0.001). Autocorrelation between these predictor variables did not disrupt the models, however.

Natural space availability was socially patterned: garden access was significantly more common for those from the least deprived neighbourhoods (χ^2^ = 82.5, p < 0.001) and most educated households (χ^2^ = 37.1, p < 0.001). Children from the least deprived neighbourhoods also had significantly more total natural space (t = 8.3, d.f. = 1395, p < 0.001), and significantly less public park space (t = −5.0, d.f. = 1395, p < 0.001), than those from the most deprived neighbourhoods. Neighbourhood natural space availability, however, did not vary with household educational attainment.

Cronbach's alpha coefficients, indicating the internal consistency of the items within each SDQ scale, were good for Total Difficulties (0.86), Emotional Symptoms (0.74), and Prosocial Behaviour (0.79), acceptable for Hyperactivity Problems (0.60) and poor for Conduct Problems (0.58), and Peer Problems (0.59).

### 3.2 Main analysis

The models’ age and age^2^ coefficients (Table 3) described the children's SDQ problem trajectories between waves four and six. Peer Problems and Total Difficulties did not change significantly, whereas Conduct Problems and Prosocial Behaviour improved over the period, and Hyperactivity and Emotional Problems worsened slightly until mean age and improved thereafter. In general, neither parks nor total natural space were associated with the SDQ domains or their change over time, although an IQR increase in total natural space was associated with Prosocial Behaviour scores 0.08 points higher (i.e., better behaviour). Having access to a garden was substantially more important than local natural space: after adjustment for both types of natural space availability those children without garden access had significantly higher scores for Hyperactivity Problems (+0.52), Peer Problems (+0.23), Conduct Problems (+0.27), and Total Difficulties (+1.15). Garden access, however, was not related to SDQ change over time. Increased screen time was related to worse outcomes for all SDQ domains except Conduct Problems.

**Table 3:** Coefficients (95% CI) for the relationship between natural space (total, parks and garden access) with SDQ domain scores and their change over time.

	Hyperactivity problems	Emotional problems	Peer Problems	Conduct problems	Total difficulties	Prosocial behaviour
FIXED PART						
Total natural space (per IQR^a^ increase)						
Total %	−0.05 (−0.14, 0.04)	−0.04 (−0.10, 0.01)	−0.04 (−0.09, 0.01)	0.01 (−0.05, 0.06)	−0.13 (−0.30, 0.05)	0.08 (0.02, 0.14)*
Total % x Age	0.30 (−0.45, 1.05)	0.23 (−0.34, 0.81)	−0.24 (−0.78, 0.30)	−0.04 (−0.56, 0.48)	0.25 (−1.18, 1.69)	0.00 (−0.68, 0.69)
Total % x Age^2^	−0.03 (−0.10, 0.05)	−0.02 (−0.08, 0.04)	0.03 (−0.03, 0.08)	0.01 (−0.05, 0.06)	−0.02 (−0.17, 0.13)	0.00 (−0.07, 0.07)
Park space (per IQR^a^ increase)						
Park %	−0.02 (−0.10, 0.05)	−0.03 (−0.07, 0.02)	−0.03 (−0.07, 0.01)	−0.02 (−0.06, 0.02)	−0.10 (−0.24, 0.04)	0.00 (−0.05, 0.05)
Park % x Age	0.42 (−0.21, 1.05)	0.10 (−0.35, 0.55)	−0.18 (−0.63, 0.28)	−0.24 (−0.70, 0.22)	0.11 (−1.12, 1.35)	0.43 (−0.15, 1.00)
Park % x Age^2^	−0.04 (−0.11, 0.02)	−0.01 (−0.05, 0.04)	0.02 (−0.03, 0.06)	0.02 (−0.02, 0.07)	−0.01 (−0.14, 0.11)	−0.04 (−0.10, 0.02)
No garden access (ref: Yes)						
No access	0.52 (0.20, 0.84)**	0.12 (−0.07, 0.31)	0.23 (0.04, 0.41)*	0.27 (0.09, 0.46)**	1.15 (0.52, 1.78)***	0.10 (−0.13, 0.33)
No access x Age	−0.07 (−2.94, 2.80)	0.59 (−1.47, 2.64)	0.52 (−1.63, 2.68)	0.19 (−1.68, 2.05)	1.19 (−4.57, 6.96)	−2.05 (−4.49, 0.39)
No access x Age^2^	0.03 (−0.27, 0.32)	−0.06 (−0.27, 0.16)	−0.04 (−0.26, 0.18)	−0.02 (−0.21, 0.18)	−0.08 (−0.68, 0.51)	0.20 (−0.06, 0.45)
Constant	6.42 (5.90, 6.94)***	2.52 (2.19, 2.85)***	2.22 (1.87, 2.56)***	3.51 (3.19, 3.84)***	14.68 (13.60, 15.77)***	6.95 (6.56, 7.35)***
Sex (ref: Male)	−0.71 (−0.85, −0.57)***	−0.02 (−0.10, 0.07)	−0.16 (−0.24, −0.08)***	−0.26 (−0.35, −0.18)***	−1.14 (−1.42, −0.87)***	0.48 (0.38, 0.57)***
Age (mean-centred)	1.32 (0.70, 1.94)***	0.47 (0.01, 0.94)*	−0.30 (−0.74, 0.15)	−0.58 (−1.02, −0.15)**	0.89 (−0.30, 2.08)	1.17 (0.61, 1.72)***
Age^2^ (mean-centred)	−0.14 (−0.20, −0.08)***	−0.05 (−0.09, 0.00)	0.02 (−0.02, 0.07)	0.04 (0.00, 0.09)	−0.12 (−0.24, 0.00)	−0.09 (−0.15, −0.03)**
Screen time (hours)	0.09 (0.04, 0.15)**	0.06 (0.01, 0.10)*	0.06 (0.02, 0.09)**	0.05 (0.00, 0.10)	0.24 (0.09, 0.40)**	−0.08 (−0.12, −0.04)**
Household education (ref: Degree)						
Vocational qualification	0.37 (0.19, 0.54)***	0.04 (−0.07, 0.14)	−0.03 (−0.14, 0.07)	0.11 (0.01, 0.21)*	0.49 (0.15, 0.82)**	0.11 (−0.01, 0.23)
Higher/Standard grade	0.52 (0.24, 0.79)***	0.07 (−0.09, 0.23)	0.10 (−0.04, 0.24)	0.24 (0.10, 0.38)**	0.91 (0.41, 1.42)**	−0.06 (−0.24, 0.12)
Other/no qualifications	0.36 (0.02, 0.71)*	0.16 (−0.09, 0.40)	0.23 (0.00, 0.46)*	0.36 (0.09, 0.63)*	1.08 (0.40, 1.77)**	−0.05 (−0.34, 0.25)
Equivalised household income (£000 s)	−0.01 (−0.02, −0.01)***	0.00 (−0.01, 0.00)	−0.01 (−0.01, 0.00)***	−0.01 (−0.01, 0.00)***	−0.03 (−0.05, −0.02)***	0.00 (0.00, 0.01)
Carer's mental health score	−0.04 (−0.05, −0.03)***	−0.03 (−0.03, −0.02)***	−0.02 (−0.02, −0.02)***	−0.03 (−0.03, −0.03)***	−0.12 (−0.14, −0.11)***	0.01 (0.01, 0.02)***
SIMD 2009 quintile (ref: Quintile 1)						
Quintile 2	0.08 (−0.16, 0.31)	0.03 (−0.11, 0.17)	0.05 (−0.08, 0.19)	0.02 (−0.12, 0.16)	0.19 (−0.27, 0.64)	−0.05 (−0.21, 0.12)
Quintile 3	0.25 (0.02, 0.47)*	0.08 (−0.06, 0.22)	0.08 (−0.06, 0.21)	0.13 (0.00, 0.27)	0.54 (0.09, 0.99)*	−0.01 (−0.17, 0.15)
Quintile 4	0.13 (−0.10, 0.35)	0.16 (0.02, 0.30)*	0.21 (0.07, 0.34)**	0.07 (−0.07, 0.20)	0.58 (0.13, 1.03)*	0.13 (−0.03, 0.29)
Quintile 5 (most deprived)	0.35 (0.11, 0.59)**	0.16 (0.01, 0.31)*	0.26 (0.12, 0.40)***	0.24 (0.10, 0.38)**	1.03 (0.56, 1.50)***	0.00 (−0.17, 0.17)
RANDOM PART						
Level 1: Wave	1.82 (1.74 to 1.89)***	1.03 (0.99 to 1.07)***	0.95 (0.91 to 0.99)***	0.87 (0.83 to 0.90)***	6.66 (6.38 to 6.94)***	1.49 (1.43 to 1.55)***
Level 2: Individual	2.93 (2.74 to 3.13)***	0.96 (0.89 to 1.03)***	0.84 (0.77 to 0.90)***	0.95 (0.89 to 1.02)***	11.53 (10.78 to 12.28)***	1.25 (1.15 to 1.34)***
Level 3: PSU	0.01 (−0.01 to 0.03)	0.00 (0.00 to 0.01)	0.00 (0.00 to 0.01)	0.00 (0.00 to 0.01)	0.03 (−0.05 to 0.11)	0.01 (−0.01 to 0.02)

* 0.01≤p < 0.05.

** 0.001≤p < 0.01.

*** p < 0.001.

a Interquartile ranges in percentage points were 16.2 for total and 6.8 for parks.

### 3.3 Stratification by sex

Boys’ SDQ scores were not related to total natural space, but some domains were related independently to park space and garden access (Table 4). Boys with garden access had reduced Peer Problems (−0.29), Conduct Problems (−0.34) and Total Difficulties (−1.18) compared to those without. Park area was independently related to boys’ Peer Problems and Conduct Problems, although effect sizes were smaller (−0.08 and −0.22 per IQR increase, respectively). In contrast, girls’ scores on some SDQ domains were related independently to total natural space and garden access but not park space. An IQR increase in total natural space around girls’ homes was associated with fewer Hyperactivity Problems (−0.15), Peer Problems (−0.08), and Total Difficulties (−0.31), and more Prosocial Behaviour (+0.14). Garden access was related to larger SDQ differences than an IQR increase in total natural space for Hyperactivity (−0.65) and Total Difficulties (−1.13). SDQ score change over time was not related to natural space or garden access for girls or boys.

**Table 4:** Coefficients for the relationship between natural space (total, parks and garden access) with SDQ domain scores, from models^a^ stratified by sex or educational attainment.

	Hyperactivity problems	Emotional problems	Peer problems	Conduct problems	Total difficulties	Prosocial behaviour
BOYS						
Total (per IQR^b^ increase)	0.03	−0.03	−0.01	0.04	0.03	0.03
Total × Age (per IQR)	−0.23	−0.12	0.02	0.17	−0.14	0.01
Total × Age^2^ (per IQR)	0.03	0.01	0.00	−0.02	0.03	0.00
Parks (per IQR increase)	−0.07	−0.03	−0.08**	−0.04	−0.22*	0.00
Parks × Age (per IQR)	0.38	−0.22	−0.33	−0.22	−0.39	0.36
Parks × Age^2^ (per IQR)	−0.04	0.02	0.03	0.02	0.04	−0.04
No garden access	0.40	0.11	0.29*	0.34*	1.18*	0.09
No garden access × Age	−1.09	0.96	0.47	1.09	1.19	−2.09
No garden access × Age^2^	0.16	−0.09	−0.04	−0.09	−0.03	0.21
GIRLS						
Total (per IQR increase)	−0.15*	−0.05	−0.08*	−0.04	−0.31*	0.14**
Total × Age (per IQR)	0.89	0.59	−0.50	−0.29	0.66	0.00
Total × Age^2^ (per IQR)	−0.09	−0.06	0.05	0.03	−0.07	0.00
Parks (per IQR increase)	0.03	−0.02	0.02	0.00	0.03	0.00
Parks × Age (per IQR)	0.47	0.49	0.00	−0.25	0.72	0.50
Parks × Age^2^ (per IQR)	−0.05	−0.05	0.00	0.02	−0.08	−0.05
No garden access	0.65**	0.13	0.15	0.20	1.13*	0.12
No garden access × Age	1.19	0.32	0.49	−1.06	1.10	−2.04
No garden access × Age^2^	−0.13	−0.04	−0.04	0.09	−0.13	0.19
LOW EDUCATION HOUSEHOLDS						
Total (per IQR increase)	−0.04	−0.06	−0.08*	−0.01	−0.18	0.06
Total × Age (per IQR)	0.80	0.59	−0.23	0.17	1.33	−0.12
Total × Age^2^ (per IQR)	−0.08	−0.06	0.02	−0.01	−0.13	0.01
Parks (per IQR increase)	−0.01	−0.04	−0.02	0.01	−0.06	−0.03
Parks × Age (per IQR)	0.41	0.06	−0.11	−0.18	0.20	0.61
Parks × Age^2^ (per IQR)	−0.05	0.00	0.01	0.02	−0.02	−0.06
No garden access	0.56**	0.11	0.19	0.26*	1.11**	0.09
No garden access × Age	−0.90	−0.13	−0.01	0.30	−0.76	−1.89
No garden access × Age^2^	0.11	0.02	0.01	−0.03	0.11	0.18
HIGH EDUCATION HOUSEHOLDS						
Total (per IQR increase)	−0.07	−0.02	0.03	0.03	−0.03	0.12*
Total × Age (per IQR)	−0.63	−0.36	−0.28	−0.39	−1.68	0.21
Total × Age^2^ (per IQR)	0.07	0.04	0.03	0.04	0.18	−0.02
Parks (per IQR increase)	−0.04	−0.02	−0.05	−0.06	−0.17	0.05
Parks × Age (per IQR)	0.20	0.12	−0.34	−0.39	−0.43	0.15
Parks × Age^2^ (per IQR)	−0.02	−0.01	0.04	0.04	0.04	−0.02
No garden access	0.40	0.09	0.24	0.27	1.00	0.20
No garden access × Age	5.46	5.47*	3.41	1.16	15.81**	−3.98
No garden access × Age^2^	−0.56	−0.55*	−0.31	−0.11	−1.56*	0.37

* 0.01≤p < 0.05.

** 0.001≤p < 0.01.

a Adjusted for age, age^2^, sex, screen time, household educational attainment, household equivalised income, carer's mental health, and neighbourhood deprivation.

b Interquartile ranges in percentage points were 16.2 for total and 6.8 for parks.

### 3.4 Stratification by household education

**Fig. 1:**
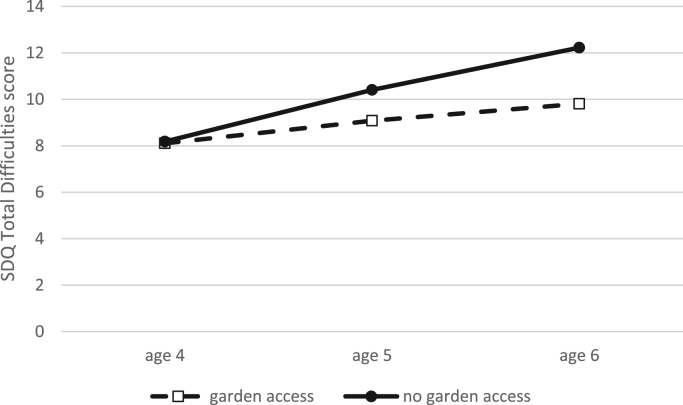
The predicted trajectory of SDQ Total Difficulties score change between ages 4 and 6 for children from high education families, with and without garden access.

An IQR increase in total natural space was associated with fewer Peer Problems (−0.08) for children from low education households, and better Prosocial Behaviour (+0.12) for those from high education households (Table 4). Not having access to a garden was associated with significantly higher levels of Hyperactivity (+0.56), Conduct Problems (+0.26), and Total Difficulties (+1.11) for children from low education but not high education households. SDQ score change over time was unrelated to natural space for either group, but garden access was related to non-linear change in Emotional Problems and Total Difficulties over time for children from high education households. As an example, Fig. 1 shows that the Total Difficulties scores of children from high education families without garden access (N.B., only 2% of the group, n = 66) worsened at a faster rate than for those with garden access.

### 3.5 Sensitivity analyses

The sensitivity analyses broadly confirmed the main results. Again, for most SDQ domains there was no association with parks or total natural space, although an IQR increase in park area was related to a 28% reduction in likelihood of abnormal Emotional Problems (OR 0.72). As in the main analyses, garden access was important: children without access to a garden were more likely to have abnormal Hyperactivity Problems (OR 2.60), and Conduct Problems (OR 1.73).

## 4 Discussion

In our study of 4–6 year old children in urban Scotland we found that certain groups with more park or total natural space around their homes had slightly better social, emotional and behavioural outcomes, compared to those with less. In contrast, having access to a garden was related to sizeable mental health benefits (particularly for Hyperactivity), on a par with the advantage apparent for girls over boys, children from degree-educated households over those from households with no educational qualifications, or a £20,000 to £50,000 increase in equivalised household income. Change over time, however, was not related to neighbourhood natural space or garden access (with one exception), suggesting that any beneficial influence had already occurred by age 4. The findings suggest that neighbourhood natural space may have a modest relationship with social, emotional and behavioural difficulties for young children, and that private natural spaces may enable the most beneficial experiences for this age group.

We did not hypothesise about the relative importance of public versus private natural space for 4–6 year olds, given the equivocal evidence (see Introduction). Play opportunities are crucial for child development, but these can be provided by public parks, other public natural spaces, or private gardens. Indeed, developmental benefits for 4–6 year olds have been reported for public parks (Balseviciene et al., 2014) and for private gardens (Flouri et al., 2014), but the two have not previously been investigated jointly. In our Scottish study, public parks were only related to improved mental health outcomes for boys (Peer Problems and Total Difficulties, with an independent significant association for private gardens), whereas private garden access was related to some improved outcomes for the whole sample, girls, boys, and those from low education households. Having access to a private garden was more frequently and strongly linked to improved mental health outcomes (Hyperactivity, Peer Problems, Conduct Problems, Total Difficulties) than increased availability of neighbourhood natural space. Carers of 4–6 year olds may be more inclined to allow relatively unsupervised play in a private garden than in public natural space, enabling more beneficial nature experiences to accrue in private settings. Prosocial Behaviour, however, was related to neighbourhood natural space (total) but not access to a private garden. This suggests that public spaces have an important role in facilitating socially-beneficial interactions, perhaps because children and adults outside of the child's immediate family and friends are more likely to be encountered.

Differences were found between boys and girls: parks appeared to be of particular importance for boys, while other natural spaces – such as amenity areas or playing fields – may be just as important for girls’ mentally-stimulating play. Compared with girls, boys in this age group engage in more active play when outdoors (Pate et al., 2013), and parks perhaps offer better safe opportunities for active play than other natural spaces. Furthermore, better social outcomes (fewer Peer Problems and/or more Prosocial Behaviour) were found for all groups with more natural space (total and/or parks) around their homes, again suggesting the importance of neighbourhood natural space in facilitating beneficial social interactions for children.

The relationship between garden access and SDQ outcomes differed by household educational status. Children from low-education households had worse outcomes (Hyperactivity, Conduct Problems and Total Difficulties) across the study period if they had no garden access, but garden access was not related to change in these outcomes over time, suggesting that any beneficial influence had already occurred by age 4. In contrast, the Emotional Problems and Total Difficulties scores of children from high-education households without garden access worsened at a faster rate than the scores of those with garden access. Children from low-education households had significantly less natural space in their neighbourhoods, so having a garden may have compensated for this in their earliest development. The results suggest the importance of garden access for social, emotional and behavioural development of children from higher educational status households becomes apparent at a later stage (i.e., after 4 years). We found no evidence that the beneficial relationships between natural space and SDQ outcomes were stronger for lower socioeconomic status children, in contrast to studies that suggest green space can buffer against the deleterious influences of social disadvantage on health (Mitchell et al., 2015).

Our study found that a high proportion of children in urban Scotland have sole or shared access to a private garden; in countries where this is not the case the potential implications for childhood development should be especially considered. Therefore, further research is needed to understand the implications of access to different forms of natural space in a range of national contexts.

In the whole-sample analyses, and those for all groups except children from high income households, we found no evidence that natural space was related to change in the SDQ outcomes between 4 and 6 years of age. The absence of such relationships suggests that any beneficial influence of park space, total natural space, or garden access had already occurred by age 4. In England, Flouri et al. (2014) also found that neighbourhood green space was unrelated to change in SDQ problem domains over time, for 3–7 year olds.

Across the whole sample in our study, neighbourhood natural space was only related to Prosocial Behaviour, whereas studies with older children have suggested whole-sample benefits of neighbourhood greenness or proximity to public green space for Peer Problems, Hyperactivity, and Total Difficulties (Amoly et al., 2014, Markevych et al., 2014). Jointly, therefore, the evidence suggests that individual and household factors (including garden access) are of key importance for the development of young children, while neighbourhood natural space may become more important as children age. The birth cohort analysed here has been followed up at ages 8 and 10, making it possible in future to investigate whether the natural environment has become more important to the children's social, emotional and behavioural development over time.

Independently of neighbourhood natural space and garden access, children with higher screen time had worse outcomes for all SDQ domains (except Conduct Problems). Strong evidence has linked early childhood television exposure with subsequent attentional problems and social disengagement (Christakis et al., 2004, Sigman, 2012). One suggested mechanism is that screen time reduces social interaction that helps children develop social and emotional skills (Sigman, 2012) – encouraging greater interaction with nature could capitalise on the benefits of nature experiences, while reducing the harms from screen use (Louv, 2005).

Our study is the first in Scotland to investigate the role of natural space in early child development, and builds upon previous research by studying younger children, using natural space measures specific to the child's home location, and applying a longitudinal approach. We used a widely-validated measure of child development – the SDQ – which has proven sensitivity for young children (Theunissen et al., 2015). We also took steps to guard against residual confounding by checking for associations with a health outcome not expected to be related to natural space. In a model fully adjusted for covariates, we found that children with more natural space (total or parks) or garden access were no more or less likely than others to have fillings, and concluded that residual confounding was not a significant issue in our models.

Certain limitations must also be acknowledged. First, our sample was unlikely to be representative of urban children in Scotland because it was subject to attrition and selection bias. Lower socioeconomic status families are less likely to continue participating in a longitudinal survey. Also, wave four and six observations were omitted if the address was different from wave five, which will have disproportionately affected low socioeconomic status households, because these were significantly more likely to have moved (results not shown). We adjusted models for household and area socioeconomic status to address this issue. Second, the SDQ was completed by the child's carer, hence was subjective. Nonetheless the reliability of the SDQ in detecting psychosocial problems has been reported elsewhere (Theunissen et al., 2015). Third, we hypothesised that natural spaces would benefit children because of the opportunities they offer for play, but our natural space measure captured quantity rather than usage. Also we could not capture natural space quality, which could be an additional influence on usage and health outcomes. Hence, finding any significant results with our crude measure of quantity is notable. Finally, our analyses were correlational, hence we were unable to prove that better outcomes in 4 to 6 year olds were caused by greener neighbourhoods or garden access.

## 5 Conclusions

We have enhanced the scarce evidence base about the role of urban natural space in childhood development, producing the first country-wide analysis to date. Our work suggests that natural space, particularly in the form of private gardens, contributes to better social, emotional and behavioural outcomes for 4–6 year old children in urban Scotland. Neighbourhood natural space may have an important role in facilitating the beneficial social interactions of young children. We found little evidence that neighbourhood natural space or garden access influenced the trajectory of developmental change between 4 and 6 years, suggesting that any beneficial influences had occurred at younger ages. Given the growing prevalence of childhood social, emotional and behavioural problems, and their implications for health in later life, our findings have public health importance. Urban planning policies that ensure children have nearby access to nature could help improve children's development. Further longitudinal studies are required to establish the mechanisms underlying the associations found, and to investigate whether the importance of neighbourhood natural space increases with age.

## Ethical approval

Wave one of the Growing Up in Scotland study was subject to medical ethical review by the Scotland ‘A’ MREC committee (application reference: 04/M RE 1 0/59). Subsequent annual waves were reviewed via substantial amendment submitted to the same committee.

## Competing financial interests declaration

The authors declare that they have no competing financial interests.
